# Association between haptoglobin, hemopexin and mortality in adults with sepsis

**DOI:** 10.1186/cc13108

**Published:** 2013-11-14

**Authors:** David R Janz, Julie A Bastarache, Gillian Sills, Nancy Wickersham, Addison K May, Gordon R Bernard, Lorraine B Ware

**Affiliations:** 1 Department of Medicine, Division of Allergy, Pulmonary and Critical Care Medicine, Vanderbilt University School of Medicine, T-1218 MCN, Nashville, TN 37232-2650, USA; 2 Department of Surgery, Vanderbilt University School of Medicine, Nashville, TN, USA; 3 Department of Pathology, Microbiology and Immunology, Vanderbilt University School of Medicine, Nashville, TN, USA

## Abstract

**Introduction:**

Plasma levels of cell-free hemoglobin are associated with mortality in patients with sepsis; however descriptions of independent associations with free hemoglobin and free heme scavengers, haptoglobin and hemopexin, are lacking beyond their description as acute phase reactants. We sought to determine the association of plasma levels of endogenous free hemoglobin and haptoglobin and hemopexin with in-hospital mortality in adults with sepsis.

**Methods:**

We conducted a retrospective observational study of a total of 387 critically ill patients with sepsis in multiple intensive care units in an academic tertiary care hospital. Measurements of plasma haptoglobin and hemopexin were made on blood drawn within 24 hours of intensive care unit admission. The primary outcome was the association between plasma haptoglobin and hemopexin with in-hospital mortality.

**Results:**

Survivors had significantly higher plasma haptoglobin concentrations (median 1234 μg/ml, interquartile range (IQR) 569 to 3037) and hemopexin concentrations (616 μg/ml, IQR 397 to 934) measured on enrollment compared to non-survivors (haptoglobin 750 μg/ml, IQR 404 to 2421, *P* = 0.008; hemopexin 470 μg/ml, IQR 303 to 891, *P* = 0.012). After controlling for potential confounders including cell-free hemoglobin concentration, patients with higher haptoglobin concentrations were significantly less likely to die in the hospital (odds ratio (OR) 0.653, 95% CI 0.433 to 0.984, *P* = 0.042), while the same association was not seen with hemopexin (OR 0.53, 95% CI 0.199 to 1.416, *P* = 0.206). In a subgroup analysis, the association between increased haptoglobin and hemopexin and decreased risk of mortality was no longer significant when analyzing patients with no detectable cell-free hemoglobin (*P* = 0.737 and *P* = 0.584, respectively).

**Conclusion:**

In critically ill patients with sepsis, elevated plasma levels of haptoglobin were associated with a decreased risk of in-hospital mortality and this association was independent of confounders. Increased haptoglobin may play a protective role in sepsis patients who have elevated levels of circulating cell-free hemoglobin beyond its previous description as an acute phase reactant.

## Introduction

Sepsis is a common condition, affecting over 700,000 adults per year in the United States with an associated mortality rate approaching 30% [1,2]. Sepsis is associated with damage to the red blood-cell membrane that results in cell lysis and release of cell-free hemoglobin into the circulation [3-6]. In humans, levels of plasma cell-free hemoglobin have been associated with poor clinical outcomes including acute kidney injury [7,8], myocardial infarction, and death [9]. Morbidity may be mediated in these conditions by the ability of cell-free hemoglobin to scavange nitric oxide in various vascular beds [10-14], damage the vascular endothelium [15], activate neutrophils [16], and oxidize lipid membranes via redox cycling [7,17]. We [18] and others [19] have recently described the presence of circulating cell-free hemoglobin in the majority of adults with sepsis; levels of cell-free hemoglobin were independently associated with poor clinical outcomes.

Haptoglobin and hemopexin are proteins produced by the liver that function, respectively, to scavenge cell-free hemoglobin and its byproduct, cell-free heme. To this end, both haptoglobin and hemopexin have been shown to have significant antioxidant properties in both animals and humans [20,21]. Additionally, in an animal model of red blood-cell transfusion with increased levels of cell-free hemoglobin, supplementation with haptoglobin attenuated the development of vasoconstriction, endothelial damage, and kidney injury [15]. In animal models of sepsis, supplementation with haptoglobin or hemopexin decreases biomarkers of inflammation [22], reduces the incidence of acute lung injury [23], improves organ function, and decreases mortality [24].

Plasma levels of haptoglobin and hemopexin have been previously described to increase in children and adults with sepsis [25-29]. Often regarded as an acute-phase reactant in response to physiologic stress, haptoglobin levels have been utilized in algorithms to aid in the diagnosis of sepsis [25,28]; however evidence to suggest that haptoglobin and hemopexin levels are associated with clinical outcomes in patients with sepsis beyond their properties as acute-phase reactants is lacking. Specifically, it is unknown whether haptoglobin and hemopexin levels are only a marker of illness or if elevations in sepsis have a protective function and are associated with improved outcomes independent of severity of illness and cell-free hemoglobin levels.

We conducted a retrospective cohort study to test the hypothesis that higher plasma concentrations of haptoglobin and hemopexin in adults with sepsis are associated with a decreased risk of in-hospital mortality, independent of severity of illness and cell-free hemoglobin levels.

## Materials and methods

### Patients

The study population consisted of 400 consecutive patients who were prospectively enrolled in the Validating Acute Lung Injury Markers for Diagnosis (VALID) study [30], had the enrollment diagnosis of sepsis as defined by the consensus definition [31], and who all had previous measurements of cell-free hemoglobin [18]. After approval by the Vanderbilt Institutional Review Board, patients who were ≥18 years old and who were admitted to Vanderbilt ICUs for at least two days were enrolled. Informed consent was obtained from patients or their surrogate decision-maker; however if neither were able or available to consent, the Institutional Review Board approved a waiver of consent given that this study posed minimal risk to participants. Plasma samples were obtained on all patients within 24 hours of ICU admission. Blood was preferentially drawn through a central venous catheter before using peripheral venous access or a peripheral blood draw in an effort to minimize hemolysis. All blood samples were immediately cooled, centrifuged at 3,000 rpm for 10 minutes, and plasma was frozen at −80°C.

### Inclusion/exclusion criteria

All 400 patients with sepsis who were consecutively enrolled were included in the analysis if they had plasma available within 24 hours of ICU admission and the sample did not appear grossly hemolyzed.

### Measurements

Plasma drawn within 24 hours of ICU admission was used for all measurements, which was the same plasma that was previously used to measure cell-free hemoglobin [18], and had been thawed and refrozen at −80°C once, prior to haptoglobin and hemopexin measurement. Haptoglobin and hemopexin were measured in duplicate by commercially available ELISA (Abcam® Haptoglobin and Hemopexin Human ELISA Kits, Cambridge, MA, USA).

### Statistical analysis

The primary analysis for this cohort was in-hospital mortality in relation to plasma haptoglobin and hemopexin levels. The secondary analysis was the association between haptoglobin and hemopexin levels and in-hospital mortality after adjusting for cell-free hemoglobin levels and other variables selected a priori and known to affect levels of haptoglobin and hemopexin, along with mortality. We also analyzed the association between haptoglobin, hemopexin and mortality in patients who did not have detectable cell-free hemoglobin as well as assessing for a potential interaction between hemopexin and cell-free hemoglobin.

As the majority of the data were not normally distributed, median values with IQR are presented for continuous variables and frequencies for categorical variables. Univariate analyses of continuous variables were conducted using Wilcoxon’s rank-sum test and Fisher’s exact test for categorical variables. We developed multivariable logistic regression models to analyze the risks of in-hospital mortality using known risk factors for poor outcomes and reduced haptoglobin and hemopexin levels, including measured levels of cell-free hemoglobin. Given that haptoglobin, hemopexin, and cell-free hemoglobin values are not normally distributed in these patients, we planned a priori to log-transform these variables when used in regression analyses. IBM® SPSS® Statistics (version 19.0, Chicago, IL, USA) was used for statistical analysis; a two-sided significance level of 0.05 was used for statistical inference.

## Results

### Clinical characteristics

Of the 400 patients with sepsis, 13 did not meet inclusion criteria due to signs of gross hemolysis during blood processing or due to missing plasma samples. The remaining 387 patients were used for the primary analyses. This patient population consisted of medical (72.1%), surgical (21.4%), trauma (3.4%), and cardiovascular (3.1%) ICU patients. Table 1 compares characteristics between survivors and non-survivors. Survivors were significantly younger, had lower severity of illness scores measured by Acute Physiology and Chronic Health Evaluation (APACHE II), were less likely to have chronic liver disease, and had lower cell-free hemoglobin levels. The median plasma haptoglobin level in all patients measured on enrollment was 1132 μg/ml, IQR 508 to 2,890 and the median plasma hemopexin level was 591 μg/ml, IQR 383 to 925.

**Table 1:** Baseline characteristics

**Characteristic**	**Overall**	**Survivors**	**Non-survivors**	***P-value***
	**n = 387**	**n = 303 (78.2%)**	**n = 84 (21.8%)**	
Age, years	57 (48, 68)	56 (47, 67)	61 (54, 70)	0.012
Men, n (%)	216 (55.8%)	164 (54.1%)	52 (61.9%)	0.217
APACHE II on enrollment	28 (22, 33)	26 (21, 32)	31 (26, 36)	<0.001
On dialysis at enrollment, n (%)	14 (3.6%)	13 (4.3%)	1 (1.2%)	0.319
PRBC transfusion, n (%)	90 (23.3%)	65 (21.5%)	25 (29.8%)	0.144
Chronic liver disease, n (%)	30 (7.8%)	16 (5.3%)	14 (16.7%)	0.002
Severe sepsis, n (%)	373 (96.4%)	290 (95.7%)	83 (98.8%)	0.319
Septic shock, n (%)	273 (70.5%)	207 (68.3%)	66 (78.6%)	0.079
ALI/ARDS, n (%)	162 (41.9%)	116 (38.3%)	46 (54.8%)	0.009
Free hemoglobin, mg/dl	20 (10, 30)	10 (10, 30)	20 (10, 40)	0.002
Haptoglobin, μg/ml	1132 (508, 2,890)	1234 (569, 3,037)	750 (404, 2,421)	0.008
Hemopexin, μg/ml	591 (383, 925)	616 (397, 934)	470 (303, 891)	0.012

Data given as median (25^th^ percentile, 75^th^ percentile) or number (percentage) of patients. APACHE II, Acute Physiology and Chronic Health Evaluation; PRBC, packed red blood cells 48 hours prior to blood draw; ALI/ARDS, acute lung injury/acute respiratory distress syndrome.

### In-hospital mortality related to haptoglobin and hemopexin concentrations

**Figure 1:**
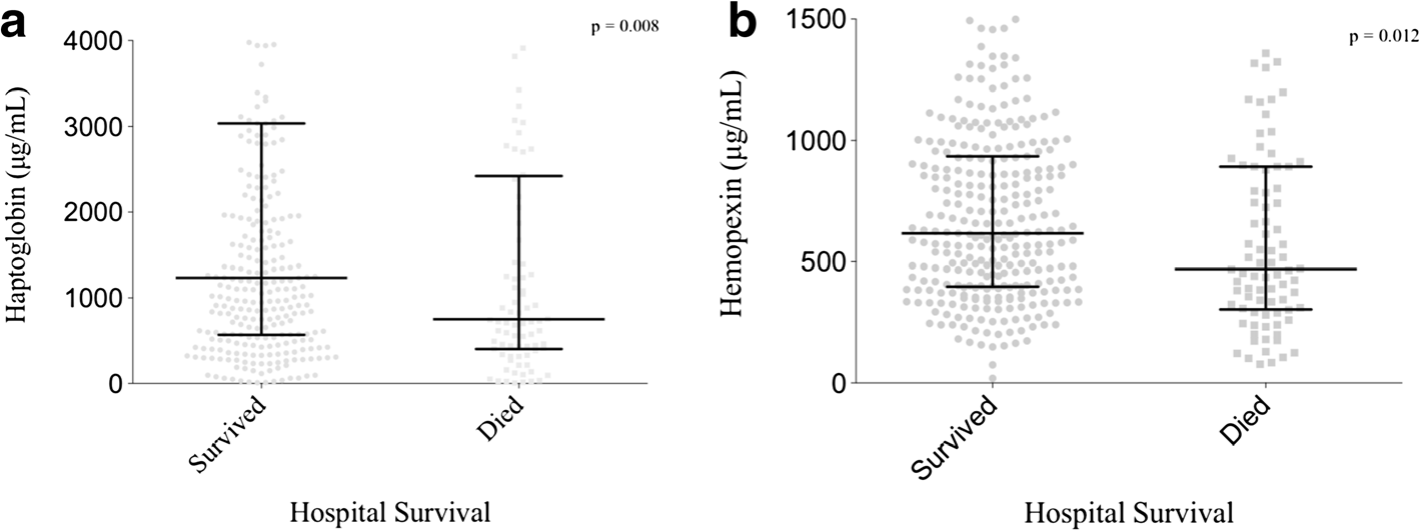
**Plasma concentrations of haptoglobin and hemopexin in relation to in-hospital mortality. (a)** Survivors had significantly higher haptoglobin (1,234 μg/dl) than non-survivors (750 μg/dl) (^*^P = 0.008). **(b)** Survivors also had significantly higher hemopexin levels (616 μg/dl) than non-survivors (470 μg/dl) (^*^P = 0.012). Values are medians (middle bold horizontal line) and IQRs (whiskers). Circles and boxes represent individual data points in each group.

**Figure 2:**
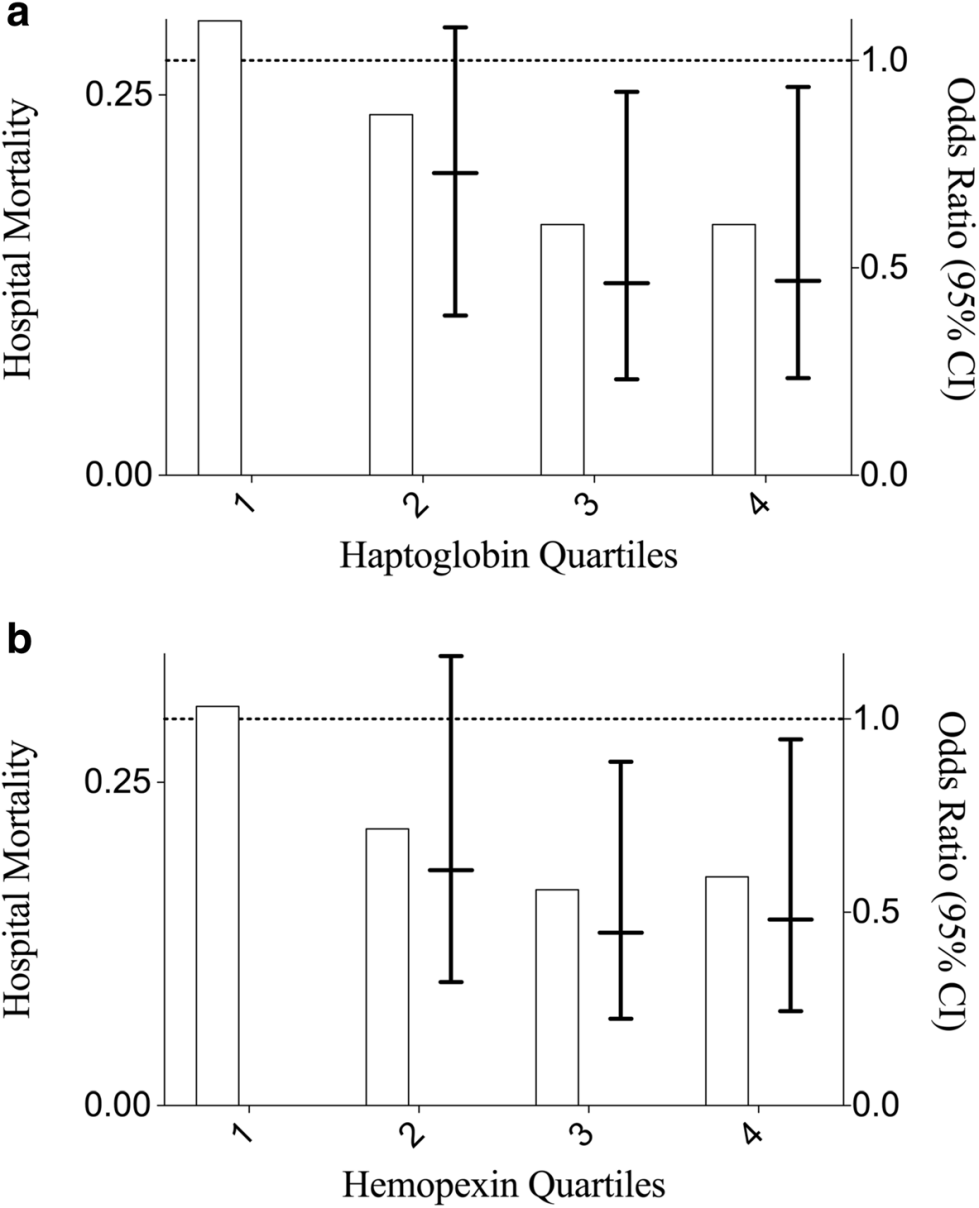
**In-hospital mortality and unadjusted odds ratios based on quartiles of haptoglobin and hemopexin.** In unadjusted analyses of the entire cohort, patients with **(a)** haptoglobin and **(b)** hemopexin in the third and fourth quartiles had significantly decreased odds of in-hospital mortality compared to patients with the lowest quartile of haptoglobin and hemopexin. Bars represent percentage of patients who died in the hospital in each quartile. Lines and whiskers represent odds ratios and 95% confidence intervals for each quartile compared to the first. Dashed line is for reference to show that the unadjusted odds of in-hospital mortality was significantly less in patients in the third and fourth quartiles of both haptoglobin and hemopexin.

Among the 387 patients in the primary analysis, 84 (21.8%) died during their hospitalization. Survivors had significantly higher haptoglobin and hemopexin concentrations (median 1,234 μg/ml, IQR 569 to 3,037 and 616 μg/ml, IQR 397 to 934, respectively) compared to non-survivors (750 μg/ml, IQR 404 to 2,421, *P* = 0.008 and 470 μg/ml, IQR 303 to 891, *P* = 0.012, respectively) (Figure 1a and b). In unadjusted analyses of the entire cohort, patients with haptoglobin and hemopexin levels in the third and fourth quartiles had significantly decreased odds of in-hospital mortality compared to patients with plasma levels in the lowest quartile of haptoglobin and hemopexin (Figure 2a and b).

Multivariable logistic regression models were used to separately examine the association between haptoglobin and hemopexin concentrations and the risk of in-hospital mortality. After controlling for age, APACHE II score, the presence of chronic liver disease, and cell-free hemoglobin levels, there was a significant decrease in risk of in-hospital mortality in patients with higher haptoglobin concentrations (odds ratio (OR) 0.653, 95% CI 0.433, 0.984, *P* = 0.042) (Table 2); however this same association was not seen with hemopexin (OR 0.53, 95% CI 0.199, 1.416, *P* = 0.206) (Table 3).

**Table 2:** Logistic regression model for in-hospital mortality in relation to plasma haptoglobin levels

**Characteristic**	**Odds ratio**	**95% confidence interval**	***P-value***
Age, years	1.022	1.004, 1.041	0.016
APACHE II score	1.075	1.040, 1.111	<0.001
Chronic liver disease	2.567	1.099, 5.999	0.029
Cell-free hemoglobin level (log, mg/dl)	2.152	1.324, 3.497	0.002
Haptoglobin (log, μg/mL)	0.653	0.433, 0.984	0.042

Odds ratios are per one year increase in age, one point increase in Acute Physiology and Chronic Health Evaluation (APACHE II) score, the presence versus absence of chronic liver disease, one log increase in cell-free hemoglobin, and one log increase in haptoglobin level.

**Table 3:** Logistic regression model for in-hospital mortality in relation to plasma hemopexin levels

**Characteristic**	**Odds ratio**	**95% confidence interval**	***P-value***
Age, years	1.022	1.004–1.041	0.017
APACHE II score	1.072	1.037–1.109	<0.001
Chronic liver disease	2.741	1.172–6.410	0.020
Cell-free hemoglobin level (log, mg/dl)	2.143	1.323–3.471	0.002
Hemopexin (log, μg/ml)	0.530	0.199–1.416	0.206

Odds ratio are per one year increase in age, one point increase in Acute Physiology and Chronic Health Evaluation (APACHE II) score, the presence versus absence of chronic liver disease, one log increase in cell-free hemoglobin, and one log increase in hemopexin level.

### Subgroup analyses in patients with and without detectable cell-free hemoglobin

**Figure 3:**
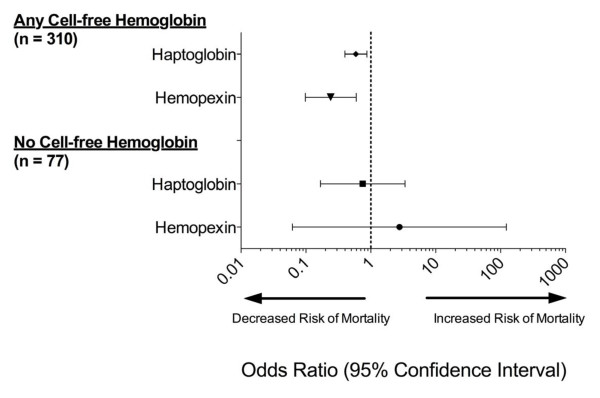
**In-hospital mortality and unadjusted odds ratios for haptoglobin and hemopexin based on the presence or absence of plasma cell-free hemoglobin.** The associated risk of in-hospital mortality was significantly lower with both increased haptoglobin and hemopexin in patients with any detectable amount of cell-free hemoglobin; however this association was no longer statistically significant in the subgroup of patients with no detectable cell-free hemoglobin.

As the potential mechanism for a protective effect of haptoglobin and hemopexin in sepsis may be their ability to scavenge cell-free hemoglobin and cell-free heme, respectively, we conducted a subgroup analysis to determine if the associated reduction in risk of in-hospital mortality was present only in patients with detectable cell-free hemoglobin. In our original cohort of 387 adults with sepsis, 310 (80.1%) had any amount of detectable cell-free hemoglobin, and 77 (19.9%) had no detectable plasma cell-free hemoglobin within 24 hours of admission to the ICU. The mortality among patients with any detectable cell-free hemoglobin was 25.2% compared to 7.8% in patients with no detectable cell-free hemoglobin (*P* <0.001). In an unadjusted analysis of patients with any amount of detectable plasma cell-free hemoglobin, increased haptoglobin levels were associated with a decreased risk of in-hospital mortality (OR 0.589, 95% CI 0.399, 0.87, *P* = 0.007), with a similar association seen with increased hemopexin (OR 0.241, 95% CI 0.098, 0.596, *P* = 0.002) (Figure 3). Among the patients with no detectable cell-free hemoglobin, the associated decreased risk of in-hospital mortality was no longer present with increased haptoglobin (OR 0.751, 95% CI 0.168, 3.364, *P* = 0.737) or hemopexin (OR 2.762, 95% CI 0.062, 122.805, *P* = 0.584).

### Assessment for interaction between cell-free hemoglobin, haptoglobin, and hemopexin

As the potential protective association that haptoglobin and hemopexin have with mortality might depend on the amount of cell-free hemoglobin present rather than confound this relationship, and given that the point estimate of increased hemopexin for the effect on in-hospital mortality increased above an odds ratio of 1.0 in the absence of cell-free hemoglobin, we assessed for interaction between cell-free hemoglobin, haptoglobin, and hemopexin. Regression models with log-transformed cell-free hemoglobin, haptoglobin, and a computed interaction term between both revealed a non-significant result (*P* = 0.968). Additionally, no statistically significant interaction was found between cell-free hemoglobin and hemopexin on in-hospital mortality (*P* = 0.581).

## Discussion

In this cohort study of critically ill patients with sepsis, there was a significant association between plasma levels of haptoglobin and in-hospital mortality. The association of haptoglobin with mortality was independent of a number of factors that may influence mortality, including plasma levels of cell-free hemoglobin; however an independent association was not seen between hemopexin and mortality. Additionally, the potential protective effect of haptoglobin against mortality in sepsis may only occur in the setting of detectable plasma cell-free hemoglobin. To our knowledge, this is the first study to describe not only the independent associations between haptoglobin and mortality in adults with sepsis, but also to study this association in the context of levels of plasma cell-free hemoglobin.

Past human studies of haptoglobin and hemopexin have focused on their properties as acute-phase reactants and as a response to the underlying inflammation associated with sepsis [25-28]. However, recent animal studies of haptoglobin supplementation for treatment of increased cell-free hemoglobin in sepsis [22-24] have created new interest in these biomarkers as potential endogenous protectants against morbidity and as well as potential therapeutics in humans with sepsis. Haptoglobin and hemopexin are endogenous scavengers of cell-free hemoglobin and cell-free heme, respectively, and have been shown in animals and humans to attenuate oxidant injury [20,21] and to reduce inflammation, acute lung injury, and mortality in animals with sepsis [22-24]. Cell-free hemoglobin is known to induce cell and tissue injury via oxidant injury, vasoconstriction, endothelial damage, and activation of neutrophils. Recent studies describe the presence of cell-free hemoglobin in animals [24] and humans with sepsis, with higher levels associated with poor clinical outcomes [18,19]. The previously described animal studies with haptoglobin supplementation and associated improved outcomes add further support to cell-free hemoglobin as a significant contributor to the morbidity and mortality associated with sepsis.

The current study suggests a role for haptoglobin in adults with sepsis beyond its past descriptions as an acute-phase reactant. Higher levels of plasma haptoglobin were associated with a decreased risk of mortality independent of severity of illness, chronic liver disease (which could impair haptoglobin production), and cell-free hemoglobin level. The association of haptoglobin levels with lower mortality was strongest in patients with detectable plasma cell-free hemoglobin and was not present in patients with no detectable cell-free hemoglobin. These data further support the potential injurious role of cell-free hemoglobin in the pathophysiology of sepsis and suggest that haptoglobin as an endogenous scavenger of cell-free hemoglobin may play a protective role in patients with sepsis rather than just being an acute-phase reactant. Although hemopexin levels were also associated with decreased mortality, this association did not persist after adjustment for chronic liver disease and severity of illness, suggesting that the association between lower hemopexin levels and mortality may primarily reflect higher severity of illness.

In conditions other than sepsis that are associated with increased levels of cell-free hemoglobin, such as cardiopulmonary bypass, red blood cell transfusion, extracorporeal circulation, and acute hemolysis, haptoglobin has been studied in humans as a potential therapeutic to prevent morbidity and mortality [32]. In the largest of these studies, patients undergoing cardiopulmonary bypass were supplemented with haptoglobin and had a significant reduction in markers of kidney injury compared to those patients who were not given haptoglobin [33]. Although the dose of haptoglobin varied widely, the patients studied had a heterogenous spectrum of disease states, and sample sizes were small, haptoglobin was generally well-tolerated and was associated with improvement in the primary endpoint in 10 of the 11 studies [32]. As the role of cell-free hemoglobin in the pathophysiology of sepsis is better elucidated, haptoglobin as a potential therapeutic will likely be attractive since both animal and human studies of diseases associated with increased cell-free hemoglobin have suggested a protective effect.

This study has several limitations. First, the retrospective cohort study design does not allow us to determine causation. Specifically, an independent association between increased levels of haptoglobin and decreased risk of death may only suggest, rather than prove, an attenuation of the deleterious effects of cell-free hemoglobin by haptoglobin, nor does this analysis tell us the specific role that haptoglobin may play in preventing mortality in patients with sepsis. We attempted to control for possible confounders with logistic regression models, however it is possible that there are unmeasured confounder, and haptoglobin is only a marker of severity of illness rather than a potential protective mediator in sepsis. Among the distribution of both haptoglobin and hemopexin measurements, there was considerable overlap for mortality status raising the possibility that the statistical difference found was less biologically meaningful. Additionally, hemopexin, as a scavenger of cell-free heme, was not independently associated with a protective effect against mortality in patients with sepsis, which raises the concern of an unmeasured confounder, lack of power to detect an association, or the potential lack of importance of cell-free heme in the pathophysiology of sepsis. We were not able to measure levels of cell-free heme in the current cohort. Finally, with regard to the subgroup analysis of patients with no measurable cell-free hemoglobin, the overall number of patients in this analysis was small, as was the number of non-survivors. The small number of non-survivors limited our ability to control for potential confounders in the subgroup analysis and also raises concern that we did not have the power to detect an interaction effect between cell-free hemoglobin, haptoglobin, and hemopexin.

## Conclusions

In critically ill patients with sepsis, increased plasma haptoglobin and hemopexin levels were associated with a reduction in in-hospital mortality. Additionally, the association of haptoglobin with mortality was found to be independent of a number of confounders, including severity of illness and plasma levels of cell-free hemoglobin. However, increased hemopexin was not independently associated with a protective effect against mortality. Prospective studies, including randomized trials, are needed to better elucidate the potential protective effects of endogenous and exogenous haptoglobin against the deleterious effects of cell-free hemoglobin in patients with sepsis.

## Key messages

•Increased plasma levels of haptoglobin and hemopexin measured early in sepsis are associated with decreased in-hospital mortality.

•This association remained statistically significant in regards to haptoglobin after controlling for a number of potential confounders, including cell-free hemoglobin.

•Haptoglobin and hemopexin may only provide protection in patients with sepsis in the setting of elevated cell-free hemoglobin levels.

## Abbreviations

ALI/ARDS: Acute lung injury/acute respiratory distress syndrome; APACHE: Acute physiology and chronic health evaluation; ELISA: Enzyme-linked immunosorbent assay; OR: Odds ratio; PRBC: Packed red blood cell; VALID: Validating acute lung injury markers for diagnosis.

## Competing interests

The authors declare that they have no financial or non-financial competing interests.

## Authors’ contributions

DRJ, JAB, and LBW were involved in the study design. DRJ, GS, NW, AKM, and GRB collected the data. DRJ, AB, and LBW performed the statistical analysis. DRJ drafted the manuscript and all authors participated in the revision of the manuscript. All authors made substantial contributions to the conception of the study, acquisition of data, and interpretation. All authors read and approved the final manuscript.
